# Transplantation of autologous bone marrow stem cells in patients with chronic spinal cord injury

**DOI:** 10.1186/1753-6561-8-S4-O14

**Published:** 2014-10-01

**Authors:** Ricardo dos Santos, Ticiana Larocca, Bruno Souza, Marcus Vinicius Mendonça, Milena Soares

**Affiliations:** 1 Centro de Biotecnologia e Terapia Celular, Hospital São Rafael, Salvador, Brazil; 2 Researcher at Hospital Espanhol, Salvador, Brazil

## 

Stem cells are being investigated by their potential use in regenerative medicine. There is currently a lack of effective clinical therapy for a number conditions, such as severe spinal cord injury (SCI). SCI can lead to chronic paraplegia, considered an irreversible condition. The administration of stem cells has been tested as a potential therapy for SCI. In the present study we evaluated the feasibility, safety and potential efficacy of autologous mesenchymal stem cells (MSC) transplantation in patients with chronic complete SCI.

We conducted a phase I/II, non-controlled study in 14 patients of both genders aging between 18-65 years, with ASIA A classified chronic traumatic SCI. Baseline somatosensory evoked potentials (SSEP), spinal magnetic resonance imaging (MRI) and urodynamics were assessed before and after treatment. Pain rating was performed using the McGill Pain Questionnaire and a visual analogue score scale.

Bone marrow-derived MSC were cultured and characterized by flow cytometry, cell differentiation assays and G-band karyotyping, showing morphological and phenotypic characteristics of MSC, as well as genetic stability. MSC were injected directly into the lesion following laminectomy and durotomy.

Transplantation of bone marrow derived MSC was an overall safe procedure. All of the patients were discharged within 48 hours after surgery. Only one patient developed a post-operatory complication, evolving a liquoric fistula that was treated by an additional surgical procedure.

Nine patients had improvements in urologic function. All patients displayed variable improvements in sensitivity and 8 patients developed lower limbs motor functional gains, principally in the hip flexors. All patients had variable improvements in ASIA global scores. Additionally, 6 patients had changes in the AIS score to grade B and one to grade C. A direct correlation between motor gain and lesion level and an inverse correlation between light touch gain and lesion volume were found 6 months after transplantation. One patient presented changes in SSEP 3 and 6 months after MSC transplantation.

Although there is an abundance of studies describing the natural history of neurologic functional gains during the first year post-SCI, there is a lack of data regarding the degree of neurological recovery that may naturally occur during prolonged follow up investigation. In the present study, we enrolled only patients with chronic and complete spinal cord injury (ASIA A) whom had previously been subjected to decompressive surgery and lengthy rehabilitation protocols without acquiring significant motor or sensory gains. Although this was not a controlled study, based on the patient profiles and the expected spontaneous gains, our study showed potential benefits of MSC transplantation treatment, in variable degrees of motor and sensory improvements, clinical pain measures and urodynamics parameters.

The use of MSC presents several advantages, such as isolation from bone marrow aspirates and large-scale expansion in cell culture procedures, which are feasible to be performed in autologous transplantations. We conclude that intralesional transplantation of autologous MSC in spinal cord injury patients is safe and feasible, and may provide some progressive benefits. We are currently beginning new clinical trials based on MSC therapy for SCI.

